# Selection of *Candida albicans* trisomy during oropharyngeal infection results in a commensal-like phenotype

**DOI:** 10.1371/journal.pgen.1008137

**Published:** 2019-05-15

**Authors:** Anja Forche, Norma V. Solis, Marc Swidergall, Robert Thomas, Alison Guyer, Annette Beach, Gareth A. Cromie, Giang T. Le, Emily Lowell, Norman Pavelka, Judith Berman, Aimeé M. Dudley, Anna Selmecki, Scott G. Filler

**Affiliations:** 1 Department of Biology, Bowdoin College, Brunswick, Maine, United States of America; 2 Division of Infectious Diseases, Los Angeles Biomedical Research Institute at Harbor-UCLA Medical Center, Torrance, California, United States of America; 3 Department of Medical Microbiology and Immunology, Creighton University School of Medicine, Omaha, Nebraska, United States of America; 4 Pacific Northwest Research Institute, Seattle, Washington, United States of America; 5 Singapore Immunology Network (SIgN), Agency of Science, Technology and Research (A*STAR), Singapore, Singapore; 6 School of Molecular Cell Biology and Biotechnology, Tel Aviv University, Tel Aviv, Israel; 7 Department of Medicine, David Geffen School of Medicine at UCLA, Los Angeles, California, United States of America; Stanford University, UNITED STATES

## Abstract

When the fungus *Candida albicans* proliferates in the oropharyngeal cavity during experimental oropharyngeal candidiasis (OPC), it undergoes large-scale genome changes at a much higher frequency than when it grows *in vitro*. Previously, we identified a specific whole chromosome amplification, trisomy of Chr6 (Chr6x3), that was highly overrepresented among strains recovered from the tongues of mice with OPC. To determine the functional significance of this trisomy, we assessed the virulence of two Chr6 trisomic strains and a Chr5 trisomic strain in the mouse model of OPC. We also analyzed the expression of virulence-associated traits *in vitro*. All three trisomic strains exhibited characteristics of a commensal during OPC in mice. They achieved the same oral fungal burden as the diploid progenitor strain but caused significantly less weight loss and elicited a significantly lower inflammatory host response. *In vitro*, all three trisomic strains had reduced capacity to adhere to and invade oral epithelial cells and increased susceptibility to neutrophil killing. Whole genome sequencing of pre- and post-infection isolates found that the trisomies were usually maintained. Most post-infection isolates also contained *de novo* point mutations, but these were not conserved. While *in vitro* growth assays did not reveal phenotypes specific to *de novo* point mutations, they did reveal novel phenotypes specific to each lineage. These data reveal that during OPC, clones that are trisomic for Chr5 or Chr6 are selected and they facilitate a commensal-like phenotype.

## Author summary

Opportunistic fungal pathogens commonly acquire extra copies of chromosomes that can provide a fitness benefit under acute stress such as exposure to antifungal agents but how these extra copies affect fungal life-style and interactions with their hosts is poorly understood. Here we show that in *C*. *albicans* the acquisition of specific whole chromosome trisomies during oropharyngeal infection in mice results in a commensal-like phenotype. Our data indicate that trisomies of chromosomes 5 and 6 alter several related virulence-associated traits that affect how the host recognizes and responds to *C*. *albicans* during oropharyngeal infection, thereby inducing this commensal-like phenotype. Whole genome sequencing revealed that trisomies were mostly maintained in subsequent oral infections and that *de novo* mutations that arose were not shared among strains. We hypothesize that both *in vivo* and *in vitro* phenotypes are likely the result of allelic imbalance of specific genes on the trisomic chromosomes, rather than due to whole chromosome trisomy.

## Introduction

Microbe-host interactions are highly complex. Following initial inoculation, multiple outcomes including colonization, commensalism, latency and disease are possible [1–3]. The immune status of the host is a key factor that determines the outcome of fungus-host interactions, especially for opportunistic fungi [4–6]. More recently, it has been appreciated that the genotype of the fungus also determines the outcome of this interaction. For example, in *C*. *albicans*, intra-species diversity among clinical strains results in differential modulation of fungus-host interactions [7]. Similarly, intra-species diversity of *Cryptococcus neoformans* is associated with different clinical outcomes in patients with cryptococcal meningitis [8].

*C*. *albicans* is generally a clonal organism without conventional meiosis, and therefore the mechanisms to generate genotypic and phenotypic diversity are limited. Nonetheless, genomic analysis of a panel of clinical *C*. *albicans* strains revealed that many contained large-scale genome changes such as whole and segmental chromosome (Chr) aneuploidy and loss of heterozygosity (LOH), with frequent aneuploidy of Chrs 5 and 7. Interestingly, one strain with a loss-of-function mutation in *EGF1* exhibited decreased systemic virulence and increased gastrointestinal (GI) colonization in mouse models [9]. LOH events were commonly associated with acquisition of antifungal resistance, while aneuploidies appeared transiently in a study of oral strain series. In addition, several virulence-associated traits such as adherence and filamentation differed among the 43 strains tested [10].

Large-scale genome changes, including whole Chr and segmental LOH, also arise *in vitro* as a result of exposure to environmental stress such as nutrient limitation, oxidative stress, temperature and antifungal drug exposure [11–17]. Importantly, the frequency, type and extent of genomic changes is influenced by the nature and severity of the stressor [11].

Exposure to the host clearly represents the most complex stress that *C*. *albicans* encounters, and this interaction cannot be fully replicated *in vitro*. Previously, we determined that genotypic and phenotypic diversity appears as early as 1 day post-infection during both hematogenously disseminated and oropharyngeal candidiasis (OPC) in mouse models of infection [18–20]. Genomic changes, in particular LOH, are 3 orders of magnitude more frequent *in vivo* compared to *in vitro* [18, 20, 21]. This strongly suggests that genome plasticity of the fungus may have a larger role in the fungus-host interaction than was previously appreciated. Our recent study of rapid *C*. *albicans* genome diversification during OPC identified a specific whole Chr amplification, trisomy of Chr 6 (Chr6x3), that was highly overrepresented among strains recovered from the tongues of mice after one round of infection. Chr6x3 was detected in 65% of mice and the allele combination ABB was 2-fold more common than the allele combination AAB [20].

Here, we tested the hypothesis that Chr6 trisomy is beneficial in the oral host environment and that strains with this genotype exhibit enhanced fitness compared to the progenitor during oropharyngeal infection. Strikingly, trisomy of Chr6x3 in two strains and trisomy of Chr5 (Chr5x3) in one strain all exhibited characteristics of commensals during oropharyngeal infection—they achieved the same oral fungal burden as the diploid progenitor strain yet caused significantly less weight loss, and they elicited a significantly lower inflammatory host response. *In vitro*, all three trisomic strains had reduced capacity to adhere to and invade oral epithelial cells. Whole genome sequencing showed that trisomies were mostly maintained, while point mutations that arose *de novo* in some lineages were unique to each lineage. Although *in vitro* growth assays did not reveal phenotypes specific to *de novo* point mutations, they did reveal novel phenotypes specific to each lineage under conditions that are relevant to fungus-host interactions and virulence potential. Taken together, our results reveal that Chr5x3 or Chr6x3 clones have a commensal-like phenotype that was apparently selected during OPC infection.

## Results

### Trisomic strains show a commensal phenotype in an oropharyngeal infection model

**Fig 1:**
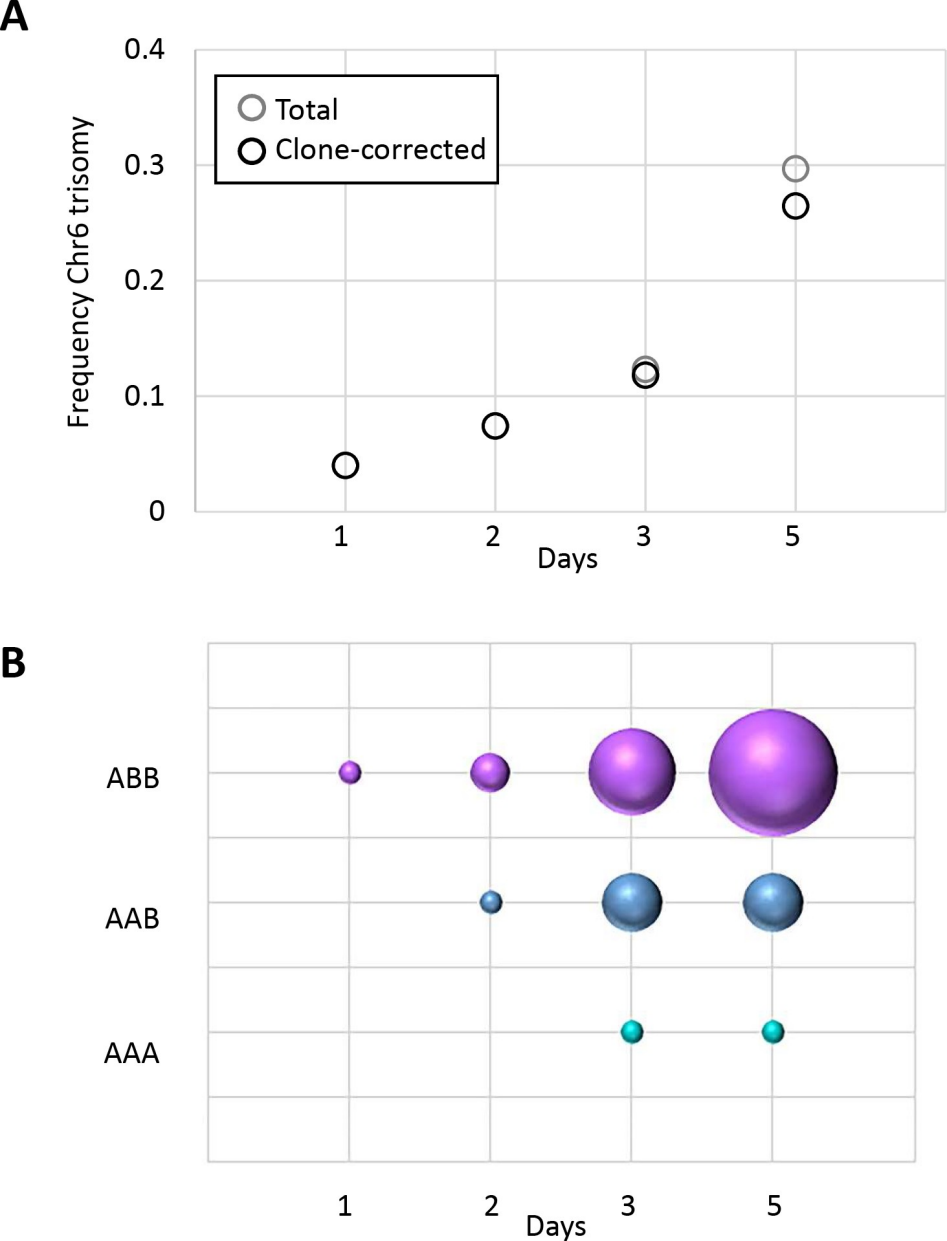
Chr6 trisomy ABB is overrepresented in isolates recovered from mice with OPC. (A) The frequency of Chr6 trisomy increases over the course of infection. (B) Among Chr6 trisomic strains, genotype ABB is the most frequent allele combination. For each genotype, symbol size is proportional to the frequency of isolation. Results are from the analysis of *C*. *albicans* colonies from 3–5 mice per time point as described in [20].

During oropharyngeal infection in mice, a specific trisomy, Chr6x3, was significantly enriched among strains and recovered from the majority of immunocompromised mice [20]. The frequency of Chr6x3 increased over the course of infection (Fig 1A) with the allele combination ABB occurring 2-fold more frequently than the AAB combination (Fig 1B), suggesting that clones with trisomy of Chr6 have a general fitness advantage during OPC and that an extra copy of allele B may be more beneficial than an extra copy of allele A in this host niche. To test this hypothesis, we selected several strains that, based on whole genome karyotypes (produced using double digest restriction-site associated DNA sequencing (ddRADseq)), had acquired single trisomies as the only change compared to the diploid progenitor, strain YJB9318. Strains AF1275 and AF1485 both had acquired Chr6x3, the former with allele combination ABB (Chr6ABB) and the latter with allele combination AAB (Chr6AAB). Each strain was originally recovered from the oropharynx of the same mouse. Importantly, these strains had not been subjected to any selection regimes (e.g., *GAL1* counterselection-induced). A third strain, AF1773, that had acquired Chr5x3 (Chr5AAB) and a small LOH on Chr1 (due to selection for *GAL1* LOH), served as control for a trisomy that did not involve Chr6. Of note, Chr5x3 was the second most common aneuploidy acquired in strains from the OPC model [20].

**Fig 2:**
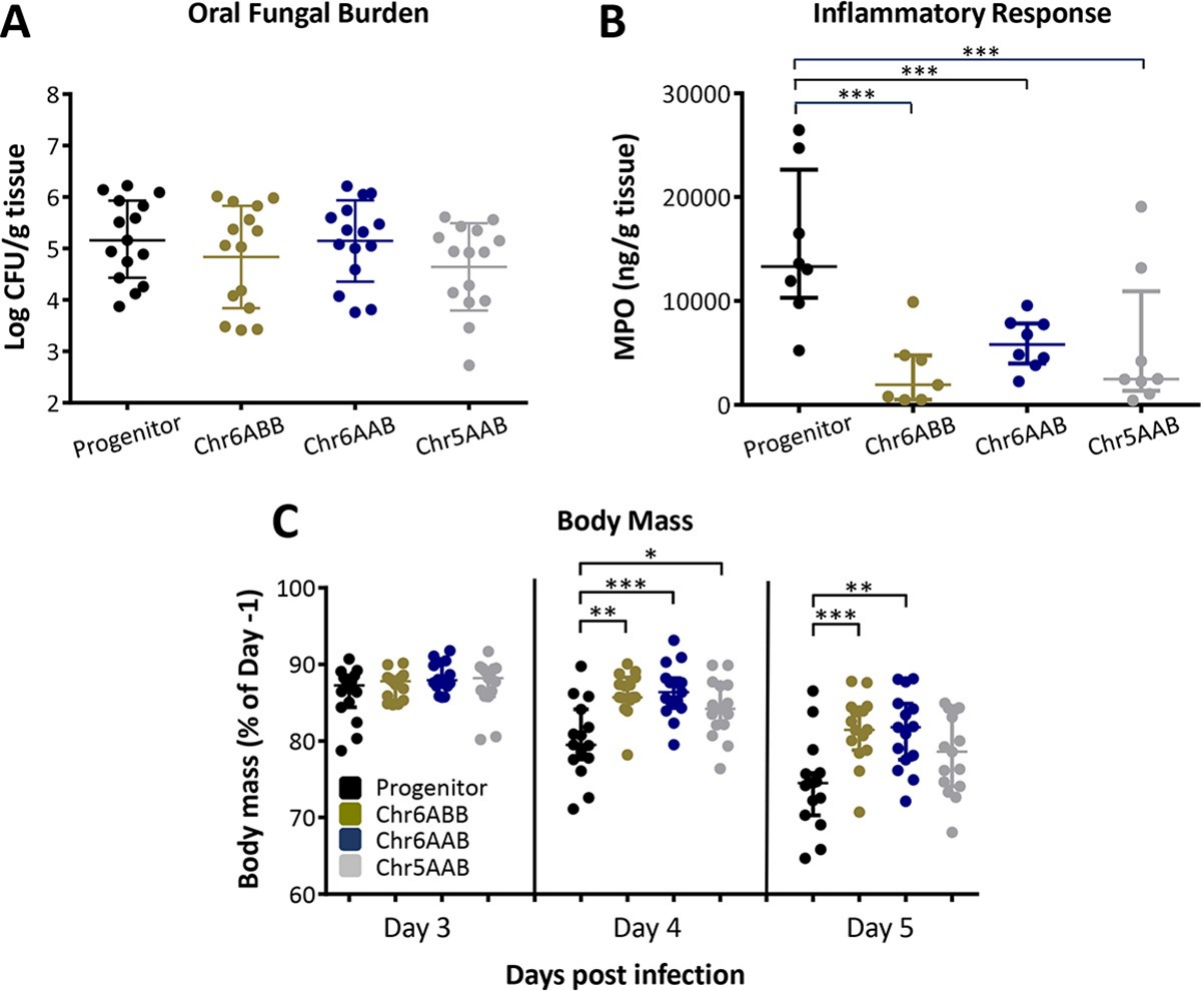
Trisomic strains exhibit a commensal-like phenotype in immunosuppressed mice. (A) Oral fungal burden of mice after 5 d of infection with the indicated *C*. *albicans* strains. (B) Myeloperoxidase (MPO) levels in the oral tissues of mice after 3 d of infection with the indicated strains. (C) Body mass of mice after 3, 4 and 5 d of infection with the indicated strains. Results in (A) and (C) are the median ± interquartile range of combined data from 2 independent experiments, each using 8 mice per strain. Results in (B) are the median ± interquartile range of data from a single experiment with 8 mice per strain. *, p < 0.05, **, p < 0.01, ***, p < 0.001 by the Wilcoxon rank sum test.

We assessed the virulence of the three trisomic strains (Chr6ABB, Chr6AAB, Chr5AAB) relative to their diploid progenitor during OPC in mice that had been immunosuppressed with cortisone acetate. Each strain was tested in 8 mice in two independent experiments for a total of 16 mice per strain. After 5 days of infection, the oral fungal burden of mice infected with all trisomic strains was similar to that of mice infected with the progenitor in terms of Log CFU per gram of tissue (Fig 2A). The inflammatory response induced by the different strains was assessed using the myeoloperoxidase (MPO) content as a marker for the accumulation of phagocytes (neutrophils and macrophages) in the oral tissues [22, 23]. Strikingly, immunocompromised mice infected with the trisomic strains had significantly lower tissue MPO levels relative to mice infected with the progenitor strain (Fig 2B). Furthermore, mice infected with the Chr6 trisomic strains lost significantly less weight than mice infected with the progenitor on days 4 and 5 post-infection, while mice infected with the Chr5 trisomic strain showed significantly less weight loss on day 4, but not day 5 post-infection (Fig 2C). Thus, the trisomic strains were able to proliferate to wild-type levels in the oropharynx, yet induced less inflammation and caused less disease, suggesting they promoted a commensal-like association with the immunosuppressed host.

### Chr6ABB behaves similar to the progenitor in an immunocompetent OPC model

**Fig 3:**
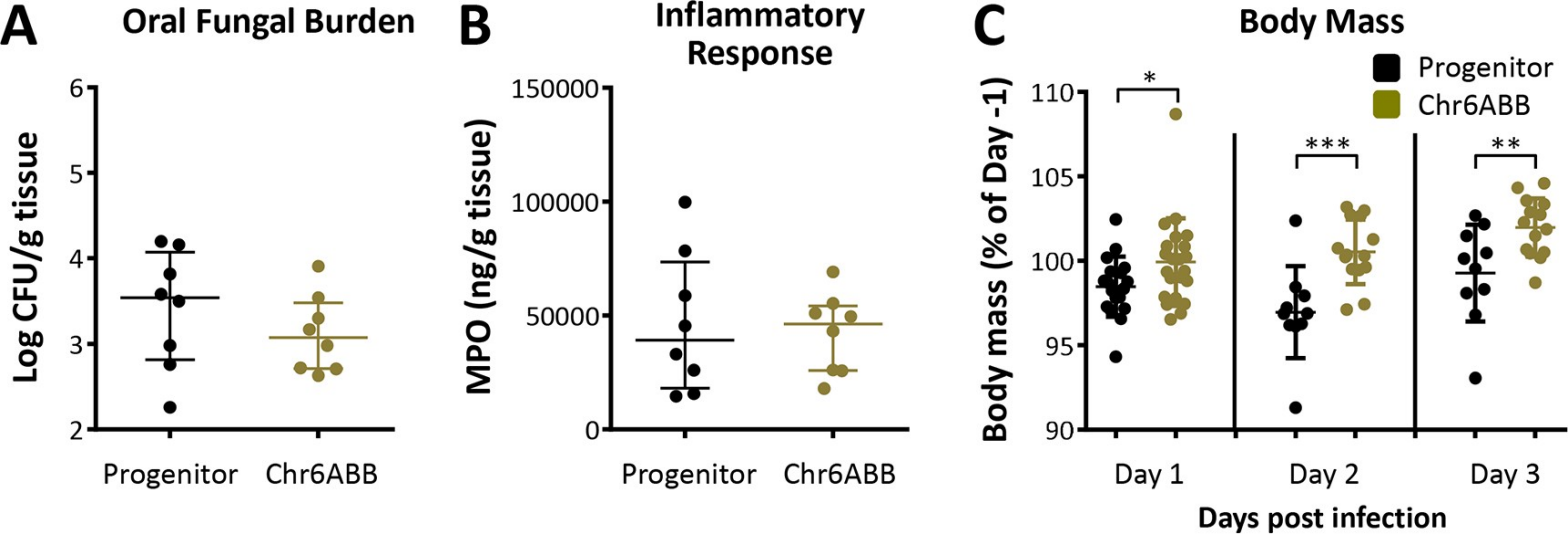
The Chr6ABB strain has a commensal-like phenotype in immunocompetent mice. (A) Oral fungal burden of immunocompetent mice after 1 d of infection with the progenitor and Chr6ABB strain. (B) MPO levels in the oral tissues of the mice after 1 d of infection with the indicated strains. (C) Body mass of the mice after 1, 2 and 3 d of infection with the indicated strains. Results in (A) and (B) are the median ± interquartile range of data from a single experiment, each using 8 mice per strain. Results in (C) are the median ± interquartile ranges of data from a single experiment with 18–22 mice per strain on d 1 and 10–14 mice per strain on d 2 and 3. *, p < 0.05, **, p < 0.01, ***, p < 0.001 by the Wilcoxon rank sum test.

Diverse clinical strains of *C*. *albicans* exhibit one of two phenotypes in the immunocompetent mouse model of OPC [7]. The commensal-like group induces a relatively weak inflammatory response and persists in the oropharynx of immunocompetent mice for a prolonged time period. Others, such as blood isolate SC5314, induce a strong inflammatory response and are cleared from the oropharynx within 2–3 days. Of note, all strains used in our study were derived from strain SC5314. To ask if Chr6x3 induces a commensal-like phenotype in immunocompetent mice, we compared the Chr6ABB strain with the progenitor strain. The oral fungal burden and MPO content of mice infected with the Chr6ABB strain was similar to that of mice infected with the progenitor (Fig 3A and 3B) after 1 day post-inoculation. By days 3 post-inoculation, the mice had cleared both strains of *C*. *albicans* from the oropharynx. Notably, the immunocompetent mice infected with the Chr6ABB strain lost significantly less weight than the mice infected with the progenitor (Fig 3C), again indicating less severe disease associated with Chr6x3.

### Infection with the Chr6ABB strain induced a weaker chemokine and IL-17A response

**Fig 4:**
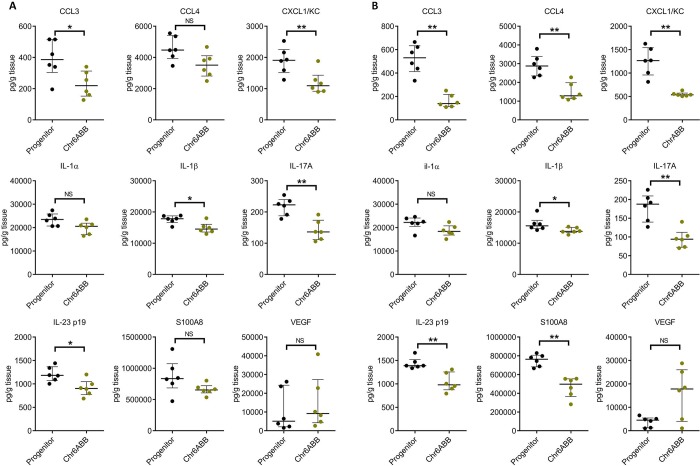
Oral infection with the Chr6ABB strain induced less production of inflammatory mediators in the oral tissues. (A) immunocompetent mice after 1 d of infection and immunosuppressed mice (B) after 5 d. Results are the median ± interquartile ranges of data from a single experiment with 6 mice per strain. *, p < 0.05, **, p < 0.01, ***, p < 0.001 NS, not significant, by the Wilcoxon rank sum test, corrected for multiple comparisons.

To further analyze the inflammatory response induced by the Chr6ABB strain, we determined the profile of chemokines, pro-inflammatory cytokines, and alarmins in the oral tissues of mice infected with this strain relative to the progenitor. In the immunocompetent mice, the Chr6ABB strain induced significantly less CCL3, CXCL1 (KC), IL-1β, IL-17A, and the p19 subunit of IL-23 in the oral tissues relative to the progenitor strain after 1 day of infection (Fig 4A). The Chr6ABB strain also induced a weaker inflammatory response in corticosteroid-immunosuppressed mice after 5 days of infection (Fig 4B). Collectively, these results indicate that the Chr6ABB strain induced an attenuated inflammatory response in both immunocompetent and immunosuppressed mice despite proliferating in the oropharynx to the same extent as the progenitor.

### The trisomic strains had reduced adherence to and invasion of oral epithelial cells, and were more susceptible to neutrophil killing

**Fig 5:**
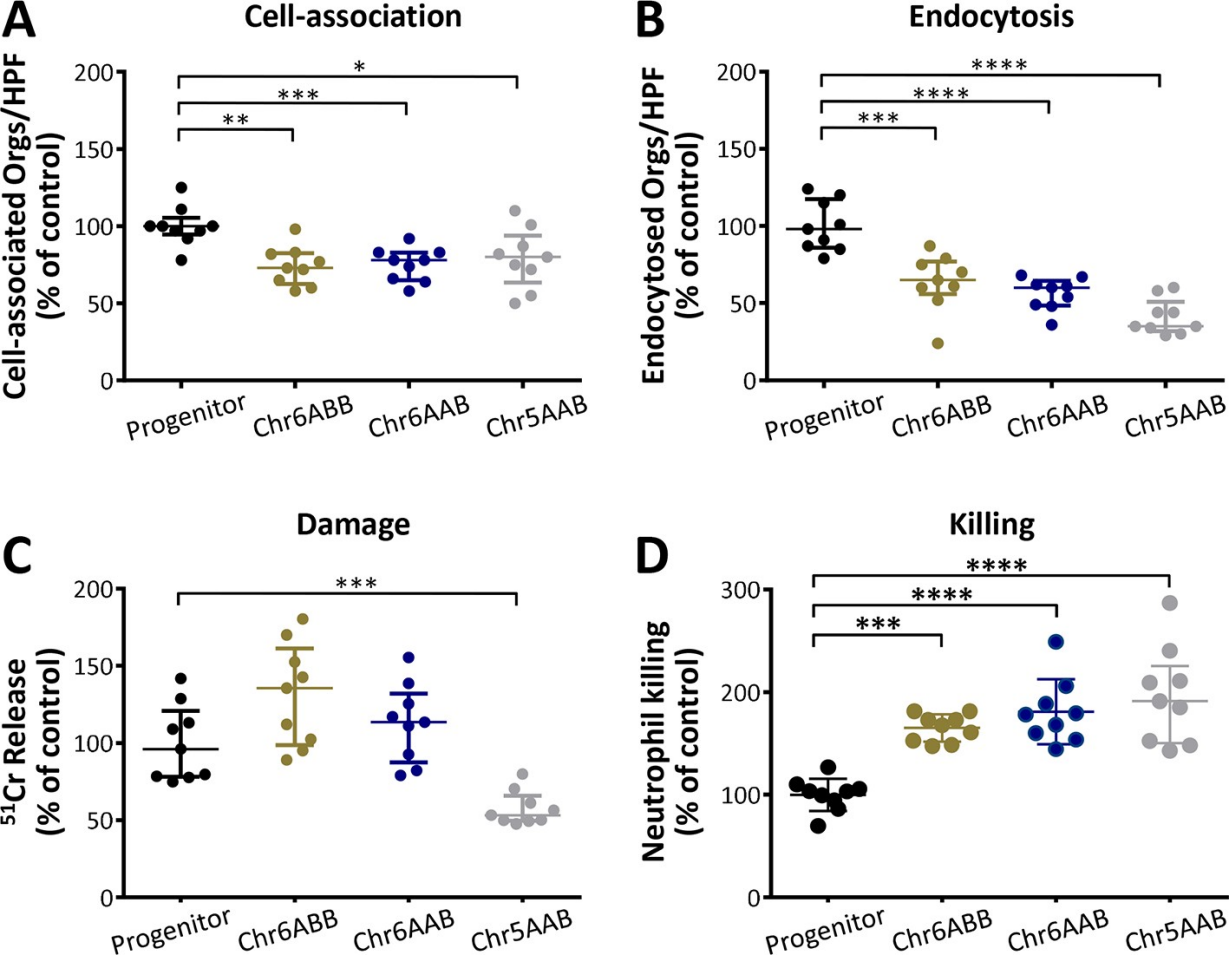
Effects of trisomy on the host cell interactions of *C*. *albicans in vitro*. (A) All trisomic strains exhibit significant defects in adherence to (A) and endocytosis by (B) oral epithelial cells. C. Only Chr5AAB shows significantly reduced damage of epithelial cells compared to progenitor D. Results are the median ± interquartile range of data from a single experiment with 6 mice per strain. *, p < 0.05, **, p < 0.01, ***, p < 0.001 ****, p < 0.0001, by the Wilcoxon rank sum test. HPF, high-power field; Orgs, organisms.

We tested the capacity of the trisomic strains to adhere to, invade, and damage oral epithelial cells, as well as their susceptibility to killing by human neutrophils, *in vitro*. The number of epithelial cell-associated organisms, a measure of adherence, was significantly reduced for the trisomic strains relative to the progenitor strain (Fig 5A). The trisomic strains also were endocytosed poorly as compared to the progenitor strain (Fig 5B). The two Chr6x3 strains (Chr6ABB and Chr6AAB) induced a similar extent of epithelial cell damage relative to the progenitor, while the Chr5x3 strain caused significantly less damage (Fig 5C). Surprisingly, all three trisomic strains were more susceptible than the progenitor strain to neutrophil killing (Fig 5D). Thus, strains with trisomy of Chr5 or Chr6 have reduced epithelial cell adherence and invasion as well as increased susceptibility to neutrophil killing *in vitro*.

### Phenotypes common and unique to each trisomic lineage

**Fig 6:**
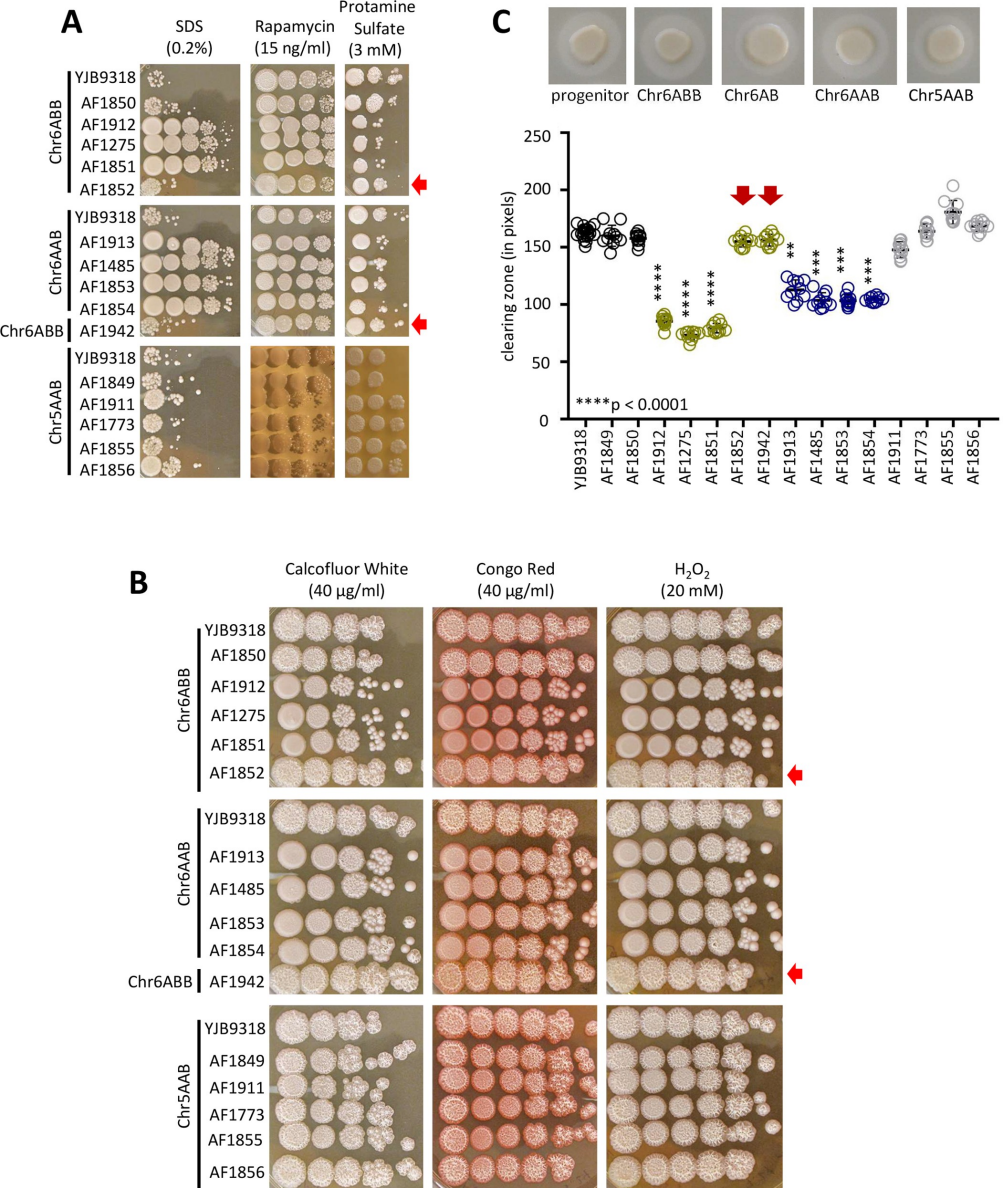
Phenotypic screening reveals lineage-specific trait changes. Note that AF1852 and AF1942, which lost one Chr6B allele, exhibit progenitor phenotypes under all conditions tested (see red arrows). A. Spot dilution assays at 37°C show differences in growth compared to the progenitor and distinct differences between Chr5 and Chr6 trisomic strains for three conditions. All images were taken on Day 3. See S1A Fig for complete growth profiles. B. Both Chr6 lineages show defects in filamentous growth at 37°C specifically under cell wall and oxidative stress. Shown are 3 representative conditions. See S1B Fig for complete profiles. C. Both Chr6 lineages show significantly lower bulk extracellular lipase and phospholipase activity compared to progenitor. Top images are representative images showing clearing zone around cell spots.

We performed growth assays by spot dilution for the original trisomic strains [20] as well as for several single colony isolates recovered after oral infection. In addition to testing 17 different growth conditions at both 30°C and 37°C, we determined lipase/phospholipase hydrolytic activity on egg yolk agar (Fig 6, S1 Fig). Under the majority of conditions, the trisomic strains grew similarly to the progenitor strain. However, the Chr6x3 strains were less susceptible than the progenitor to SDS and rapamycin, and more susceptible to protamine sulfate (Fig 6A, S1 Fig). By contrast, the Chr5AAB strain was more susceptible to rapamycin, but less susceptible to protamine sulfate. Interestingly, all three strains showed less filamentous growth that the progenitor (spots were smooth or only slightly wrinkled vs wrinkled progenitor colonies) under a variety of conditions (Fig 6B, S1 Fig). These observations were strain-specific, with Chr5AAB showing the fewest defects and Chr6ABB the most defects in filamentous growth (Fig 6B, S1 Fig). The Chr6x3 strains also had reduced extracellular lipase/phospholipase hydrolytic activity relative to the progenitor strain (Fig 6C). Importantly, strains AF1852 and AF1942, both of which lost one Chr6B allele during oral infection, exhibited progenitor phenotypes under all conditions tested (Fig 6, S1 Fig). This result strongly supports the idea that the extra copy of Chr6B was necessary and sufficient to induce the observed phenotypes in the Chr6x3 strain.

### Trisomies in Chr6 were mostly maintained during OPC

**Fig 7:**
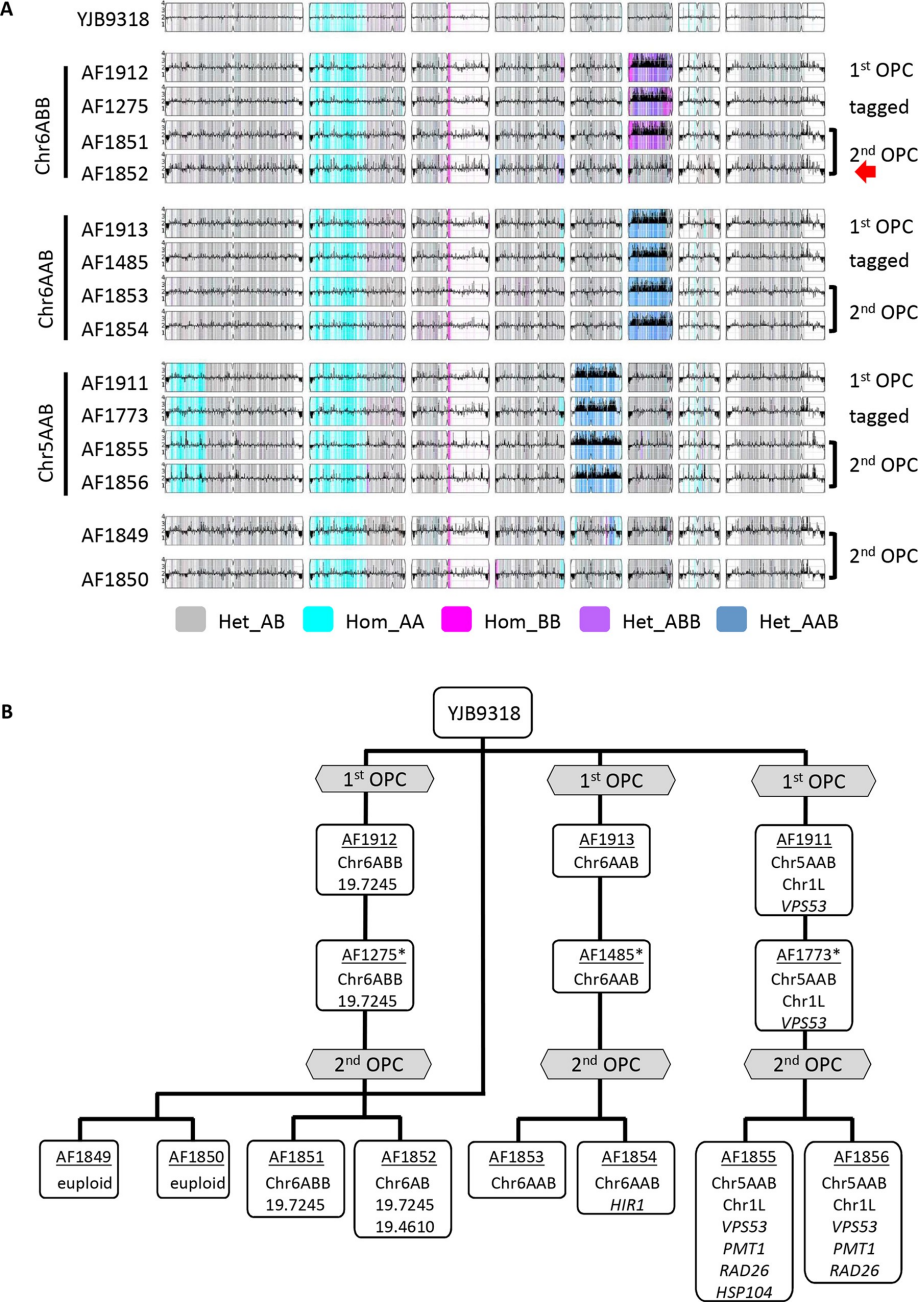
Whole genome sequence analysis of the trisomic strains. (A) The genomes of most strains were stably maintained during transformation and second oral infection. YMAPs showing the local copy number estimates for strains from which whole genome sequence data were obtained. Note that strain AF1852 had lost one copy of Chr6B. Het, heterozygous; hom, homozygous; 1^st^ OPC, original oral infection [20]; 2^nd^ OPC, strains isolated from mice in the current experiments. (B) *De novo* point mutations arose during the 2^nd^ OPC but were not shared among trisomic lineages. Diagram of the strain lineages showing the genomic changes and the SNPs in the indicated genes (see S2 Table for detailed information on SNPs); * Tagged with unique barcode at the NEUT5L locus.

Because strains with aneuploid chromosomes are often unstable, we measured strain ploidy levels both before and after OPC infection, using a qPCR approach that sampled 4 markers along each of the eight chromosomes from inoculum streaks, from single colonies of the inoculum, and from single colonies recovered from different mice after OPC. At the population level (streak), all 4 strains maintained their original karyotype over the three days of inoculum preparation (S2 Fig). However, when single colonies were analyzed, 1 of 5 colonies of the Chr6ABB strain had lost Chr6 trisomy, indicating that Chr6x3 is not very stable, and raising the possibility that the initial inoculum of this strain was a mixed population. Next, we analyzed multiple colonies from all four strains to ask if any large-scale genomic changes had occurred during infection. No novel aneuploidies were found in any of the analyzed strains (Fig 7A, S2 Fig). In total, 27% (6/22) of individuals from the Chr6ABB strain background had lost the trisomy during the course of the OPC infection experiment. Similarly, 31% (5/16) of the strains from the Chr6AAB strain background were no longer trisomic. By contrast, all post-infection strains of the progenitor and of the Chr5AAB strains had maintained their original karyotypes (see also below). Thus, Chr6x3 appears to be more unstable than Chr5AAB and thus likely requires positive selection for its maintenance in the OPC model.

### Trisomic strains acquired relatively few *de novo* point mutations during OPC, and these were not conserved

SNPs, INDELS, LOH and CNVs were identified in the post-OPC isolates using Illumina whole genome sequencing (see Fig 7A). Maintenance and loss of aneuploid chromosomes, detected by qPCR, were confirmed for all strains (Fig 7B). Importantly, the karyotypes of these strains were stable during transformation and during 5 days of OPC in all strains except for strain AF1852, which was a post-infection Chr6ABB strain (Fig 7B). No *de novo* large-scale LOH events were observed. We identified a total of 8 *de novo* SNPs/Indels, all of which were heterozygous; we verified each SNP by Sanger sequencing of the parent and evolved strains (Fig 7A, S1 Table). Seven of these SNPs were located in coding regions and one was intergenic (S1 Table). Thus, *de novo* mutations were identified in all three lineages, with none of them were recurrent (shared among strains). These results strongly suggest that the commensal-like phenotype was indeed due to whole Chr aneuploidies.

## Discussion

When *C*. *albicans* infects a mammalian host, it is exposed to a variety of different stressors that vary with both the anatomic niche and the duration of infection. Many stressors, such as reactive oxygen species produced by immune cells, antimicrobial peptides, and sequestration of micronutrients are generated by the host. During infection of non-sterile mucosal surfaces, the fungus must also withstand stressors generated by the competing bacterial microbiota. The magnitude of stress experienced by *C*. *albicans* at a specific infection site is likely dependent on whether the organism is growing as a commensal or a pathogen. For example, when *C*. *albicans* grows in the oropharynx as a commensal, it induces only a weak host response and is thus unlikely to experience significant oxidant stress induced by activated phagocytes. By contrast, when *C*. *albicans* overgrows and causes OPC, the massive influx of neutrophils subjects the organism to substantial oxidant stress [4, 24]. The multiplicity of stressors encountered by *C*. *albicans* necessitates that it adapts to specific host micro niches.

Aneuploidy and LOH of *C*. *albicans* generated *in vivo* is thought to enhance the survival and proliferation of the fungus within the host [18–21, 25], a hypothesis that has been tested only in a mouse model of GI colonization [21, 25]. In particular, experimental evolution in the mouse GI tract selects for *C*. *albicans* variants harboring whole-chromosome as well as segmental aneuploidy and LOH events [21, 25] and results in commensal, attenuated strains that protect their host against subsequent infection with a virulent *C*. *albicans* strain [25]. The overrepresentation of strains with Chr6x3 in our earlier study [20], despite the instability of the trisomic state, suggests that this trisomy provides fungal cells with a competitive advantage during OPC. The results presented here demonstrate that trisomies of Chr6 and Chr5 enable *C*. *albicans* to infect the oropharynx at high levels, yet causes less disease. Specifically, when immunosuppressed mice were orally inoculated with the trisomic strains, their oral fungal burden was similar to that of mice infected with the diploid progenitor strain. At the same time, the trisomic strains induced a lower inflammatory response in terms of reduced phagocyte accumulation and decreased cytokine levels, which resulted in less weight loss than in mice infected with the progenitor strain. These results suggest that trisomy in either Chr6 or Chr5 causes *C*. *albicans* to begin to assume a commensal-like phenotype.

To further explore the mechanism of the commensal-like phenotype of the trisomic strains, we analyzed the host response to OPC caused by the Chr6 ABB strain relative to the progenitor strain. In both immunocompetent and immunosuppressed mice, the Chr6 ABB strain induced lower production of multiple chemokines and pro-inflammatory cytokines. The decreased levels of CCL3 and CXCL1, which are neutrophil chemoattractants, provides an explanation for the reduced levels of the phagocyte marker MPO in the oral tissues of mice infected with the trisomic strains. Reduced levels of IL-17A and IL-23, which play key roles in the host defense against OPC [26], also suggest that the trisomic strain induced less disease during OPC and are consistent with the commensal-like phenotype of the trisomic strains.

A potential explanation for the reduced pathogenicity of the Chr6x3 and Chr5x3 strains is provided by our *in vitro* studies: trisomic isolates had reduced capacity to adhere to and invade oral epithelial cells. *C*. *albicans* expresses a multitude of adhesins that mediate the binding of the fungus to epithelial cells (reviewed in [27]). One of these adhesins is Als3, which also functions as an invasin [28, 29]. One possible explanation for the reduced adherence and invasion of the trisomic strains is that they have reduced surface expression of Als3. However, by flow cytometric analysis of hyphae stained with an anti-Als3 antibody, we found that Als3 surface expression of all three trisomic strains was similar to that of the progenitor strain (S3 Fig). These results suggest that the adherence and invasion defects of the trisomic strains are due to reduced expression of one or more adhesin(s) and invasin(s) other than Als3.

When Schonherr *et al*., [7] investigated the epithelial cell interactions of different strains of *C*. *albicans*, they found that the ones with the commensal phenotype caused less damage to epithelial cells *in vitro* than did the pathogenic strains. We found that only the Chr5 AAB strain had reduced capacity to damage the epithelial cells; both Chr6 trisomic strains induced wild-type levels of epithelial cell damage. Thus, reduction of *in vitro* epithelial cell damage did not predict the commensal-like phenotype of the Chr6 trisomic strains.

Surprisingly, all three trisomic strains had increased susceptibility to neutrophil killing, yet the oral fungal burden of mice infected with these strains was similar to that of mice infected with the progenitor strain. We speculate that the trisomic strains were able persist in the oropharynx in high numbers because of the defect in phagocyte recruitment.

Whole genome sequence analysis did not identify any common point mutations among the trisomic lineages. This result supports the concept that the trisomic state of specific chromosomes, rather than specific point mutations, resulted in the commensal-like phenotype. In a recent study, trisomy of Chr7 (Chr7x3) was found to be common in *C*. *albicans* strains after *in vivo* passage in a mouse model of GI colonization [21]. Chr7x3 arose in three different strain backgrounds and conferred a fitness (growth) advantage over the respective diploid progenitors in 1:1 *in vivo* competition experiments. Thus, the development of trisomy in specific chromosomes appears to influence the capacity of *C*. *albicans* to persist in distinct anatomic niches.

Chr6 harbors many genes important for filamentous growth, adhesion and hydrolytic enzyme production [30]. Importantly, strains that lost the extra copy of the Chr6B allele from the Chr6ABB strains reverted to the parental phenotypes. These data support the idea that the commensal phenotype is a multi-gene trait that is fostered by an extra copy of Chr6B. This allelic imbalance in the trisomic strains may also affect cell wall architecture and/or composition, which would then alter immune recognition by the host. Accordingly, the amount of exposed β-glucan on the surface of fungal cells was strongly predictive of competitive fitness in the mouse GI tract [21]. Furthermore, fungal cell wall architecture, rather than cell wall composition, determines the ability of fungi to colonize the GI tract [31]. Finally, a recent study on functional divergence of filamentous growth regulation in *C*. *albicans* found that Flo8 overexpression was sufficient to restore filamentation in a *mfg1*/*mfg1* mutant [32]. Importantly, Flo8 is located on Chr6 and *in vitro* evolution of three *mfg1*/*mfg1* lineages *in vitro* resulted in trisomy of Chr6 [32].

Taken together, our data indicate that specific whole chromosome aneuploidies alter several related virulence-associated traits that affect how the host recognizes and responds to *C*. *albicans* during oropharyngeal infection, thereby inducing a commensal-like phenotype. Because both the *in vivo* (commensal) and *in vitro* phenotypes are likely the result of allelic imbalance of specific genes on the trisomic chromosomes, rather than due to whole chromosome trisomy, it will be imperative to identify those genes that, when present in an extra copy, enhance the capacity of *C*. *albicans* to interact with the host and survive in diverse anatomic sites.

## Materials and methods

### Strains used in this study

Strains are listed in S2 Table and were stored at -80°C in 50% glycerol.

#### Ethics statement

All animal work was approved by the Institutional Animal Care and Use Committee (IACUC) of the Los Angeles Biomedical Research Institute under protocol 30842–03 (Animal Welfare Assurance Number A3330-01). The experiments were performed in accordance with recommendations in the Guide for the Care and Use of Laboratory Animals of the National Institutes of Health. The collection of blood from healthy human volunteers for neutrophil isolation was approved by the Institutional Review Board of the Los Angeles Biomedical Research Institute under protocol 30842–03 (Human Subject Assurance Number 00001992). Written informed consent was obtained from each subject prior to phlebotomy.

#### Mouse model of OPC

The pathogenicity of the *C*. *albicans* strains during OPC was determined in both immunocompromised and immunocompetent male Balb/c mice following our standard protocol [33]. When immunocompromised mice were used, cortisone acetate (2.25 mg/kg) was administered subcutaneously on days −1, 1, and 3 [33]. For inoculation, the animals were sedated with ketamine and xylazine, and a swab saturated with 10^6^ *C*. *albicans* cells was placed sublingually for 75 min. Immunocompetent mice were inoculated similarly, except that the swab was saturated with 2 x 10^7^ organisms [34, 35]. The immunocompromised and immunocompetent mice were sacrificed at 3 and 5 d and 1 and 3 d post-infection, respectively. After sacrifice, the tongue and attached tissues were harvested and divided longitudinally. One hemisection was weighed, homogenized, and quantitatively cultured. The other was embedded in paraffin, after which thin sections were prepared and then stained with periodic acid-Schiff stain (PAS).

#### Human cell line

The human oral epithelial cell line OKF6/TERT-2 was kindly provided by J. Rheinwald (Harvard University, Cambridge, MA) [36] and was cultured as previously described [28].

#### Host cell damage assay

The extent of oral epithelial cell damage caused by different *C*. *albicans* strains was determined using our previously described ^51^Cr release assay [35]. Briefly, OKF6/TERT-2 cells were grown to 95% confluence in 96-well tissue culture plates with detachable wells (Corning) and loaded with 5 μCi/ml Na_2_^51^CrO_4_ (PerkinElmer) overnight. After rinsing the cells to remove the unincorporated ^51^Cr, they were infected with 6 x 10^5^ *C*. *albicans* cells. After 7 h, the amount of ^51^Cr released into the medium and retained by the cells was determined by gamma counting. Each experiment was performed three times in triplicate.

#### Measurement of *C*. *albicans* epithelial cell adherence and endocytosis

*C*. *albicans* adherence to and endocytosis by oral epithelial cells was quantified by a differential fluorescence assay as described previously [37]. Briefly, OKF6/TERT-2 cells were grown to confluency on fibronectin-coated circular glass coverslips in 24-well tissue culture plates. They were infected with 2 x 10^5^ yeast-phase *C*. *albicans* cells per well and incubated for 2.5 h, after which they were washed, fixed, stained, and mounted inverted on microscope slides. The coverslips were viewed with an epifluorescence microscope, and the number of adherent and endocytosed organisms per high-power field (HPF) was determined, counting at least 100 organisms per coverslip. Each experiment was performed at least three times in triplicate.

For MPO analysis during OPC, the tongue homogenates from immunocompromised mice a 3 d post-infection were clarified by centrifugation, and stored at −80°C. The MPO concentration was determined using a commercial ELISA kit (Hycult Biotech).

### Measurement of cytokines in immunocompromised and immunocompetent mice

#### *C*. *albicans* Als3 surface expression

Flow cytometry was used to analyze the surface expression of Als3 on *C*. *albicans* strains, using our previously described method [28, 38]. Briefly, after fixing *C*. *albicans* cells in 3% paraformaldehyde and blocking with 1% goat serum, the cells were incubated with a rabbit polyclonal antiserum raised against the recombinant N-terminal region of Als3. Next, the cells were rinsed and incubated with a goat anti-rabbit secondary antibody conjugated to Alexa Fluor 488 (Invitrogen). Cell sorting was performed with a FACSCaliber flow cytometer (Becton, Dickinson). Fluorescence data for 10,000 cells of each strain were collected.

#### Neutrophil killing

The susceptibility of the various *C*. *albicans* strains to neutrophil killing was determined as described previously [35]. Briefly, neutrophils were strained from the blood of healthy volunteers and mixed with an equal number of *C*. *albicans* cells in RPMI 1640 medium plus 10% fetal bovine serum. After a 3 h incubation at 37°C in 5% CO_2_, the neutrophils were lysed by sonication, and the number of viable *C*. *albicans* cells was determined by quantitative culture. Each experiment was performed three times in triplicate (different donors).

#### Statistics

Data were compared by Mann-Whitney or unpaired Student’s t test using GraphPad Prism (v. 6) software. P values of < 0.05 were considered statistically significant.

#### Quantitative PCR (qPCR) to assess chromosome copy number

To check that the trisomies were maintained during inoculum preparation and throughout the course of infection we used qPCR for 4 markers along each of the 8 chromosomes to determine ploidy (see S3 Table for primer sequences). qPCR was performed on gDNA extracted from single colonies and from streaks of the inoculum and for strains recovered from the tongues of infected mice. Streaks or single colonies were transferred from original plates directly into either 750 μl (streaks) or 150 μl (single colonies) of 50% glycerol, and 100 μl were used for gDNA extraction without additional culturing. Resulting gDNA amounts were sufficient for qPCR.

#### Whole genome sequencing and Sanger sequencing

Genomic DNA was isolated with phenol chloroform, as described previously [39]. Libraries were prepared using the NexteraXT DNA Sample Preparation Kit following the manufacturer’s instructions (Illumina). Libraries were purified with AMPure XP beads (Agencourt) and library concentration was quantified using a Bioanalyzer High Sensitivity DNA Chip (Agilent Technologies) and a Qubit High Sensitivity dsDNA fluorometric quantification kit (Life Technologies). DNA Libraries were sequenced using paired-end 2 x 250 flow cells on an Illumina MiSeq (Creighton University). Copy number and allele status was visualized using YMAP [40]. Fastq files were aligned using an in-house sequence analysis pipeline [41–43] and custom Python scripts. The progenitor strain and any of the evolved [44] strains were analyzed using Mutect [45] resulting in individual output files containing *de novo* SNPs that were acquired by the evolved strains. SNP regions were validated by eye in IGV. For non-synonymous *de novo* SNPs, primer pairs were generated with Primer 3 [46] (S3 Table) to yield PCR products of about 400 bp. SNPs were confirmed by Sanger sequencing of amplified products as described [47].

#### Spot dilution assays

Strains were streaked onto YPD plates and incubated for 3 days at 30°C. Single colonies were transferred to 3 ml YPD broth and grown overnight at 30°C in a roller incubator. Cells were spun down, washed twice with PBS buffer and resuspended in 1 ml PBS. The number of cells/ml was counted using a hemacytometer and all strains were adjusted to 1 x 10^9^ CFU/ml. For each strain, five microliters of a five-fold dilution series were spotted onto YPD, YPD light (0.2% glucose), YPD supplemented with 1 M NaCl, 150 μM Farnesol, 40 or 80 μg/ml Congo Red, 40 or 80 μg/ml Calcofluor White, 0.1 or 0.2% SDS, 10, 20, 40, or 100 mM H_2_O_2_, 15 ng/ml Rapamycin, Casitone media supplemented with 4 μg/ml fluconazole, or 0.1 μg/ml caspofungin, 3 mM Protamine sulfate, RPMI supplemented with 0.2% glucose and Spider medium [48]. Four sets were prepared for each medium, and sets were incubated at room temperature, 30°C, 37°C, and 42°C, respectively, monitored for growth and colony appearance and photographed on days 2, 3 and 6.

#### Extracellular lipase/phospholipase activity

To assay lipase and phospholipase hydrolytic activity, five microliters of a 1 x 10^9^ CFU/ml stock was spotted onto egg yolk agar plates (EYA; 10% egg yolk emulsion, 1% peptone, 1.5% agar, 3% glucose, 5.73% NaCl, 0.055% CaCl_2_) [49]. Spots were allowed to dry and plates were incubated at 37°C for up to 5 days, photographed on day 5 and images were analyzed with ImageJ. For each strain, twenty measurements of the extent of the clearing around each spot were taken. To determine whether the differences in clearing were significantly different from the parental strain and between strains unpaired Student’s t tests were done using GraphPad Prism (v. 6) software. A p-value of < 0.05 was considered significant.

## Supporting information

S1 Fig Summary of phenotypic evaluation of strain lineages. Shown are the conditions for which differences in growth (A) and spot morphology (B) were seen either between the progenitor and the trisomic strain(s) or between the different trisomic strains. Data is arranged by the day the plates were scored, the incubation temperature and growth medium. Strains and their derivatives are ordered as follows: progenitor, Chr6ABB, Chr6AAB, Chr5AAB. See S1 Table for strain information.
(TIF)

S2 Fig Variable retention of trisomies suggest population heterogeneity *in vivo*. Ploidy genotypes were stably maintained for the parent and the Chr5AAB lineage but not for either Chr6 lineage. The ploidy genotype of the indicated strains was determined by qPCR for 4 markers along each of the 8 chromosomes.
(TIF)

S3 Fig Surface expression of Als3. Flow cytometric analysis of progenitor (YJB9318), AF1275 (Chr6ABB), AF1485 (Chr6AAB), AF1773 (Ch5AAB), and an *als3*Δ/Δ delete strain as negative control. The histogram shows the results of analysis of 10,000 cells per strain.
(TIF)

S1 Table Summary of confirmed high-confidence SNPs. (XLSX)

S2 Table Strains used in this study. (DOCX)

S3 Table Primers used to confirm high confidence non-synonymous SNPs. (DOCX)
