# Supplementary figures and images for: Selection of Candida albicans trisomy during oropharyngeal infection results in a commensal-like phenotype
Source: PLoS Genet. 2019 May 15;15(5):e1008137. doi: 10.1371/journal.pgen.1008137 (PMC6538192; doi:10.1371/journal.pgen.1008137)

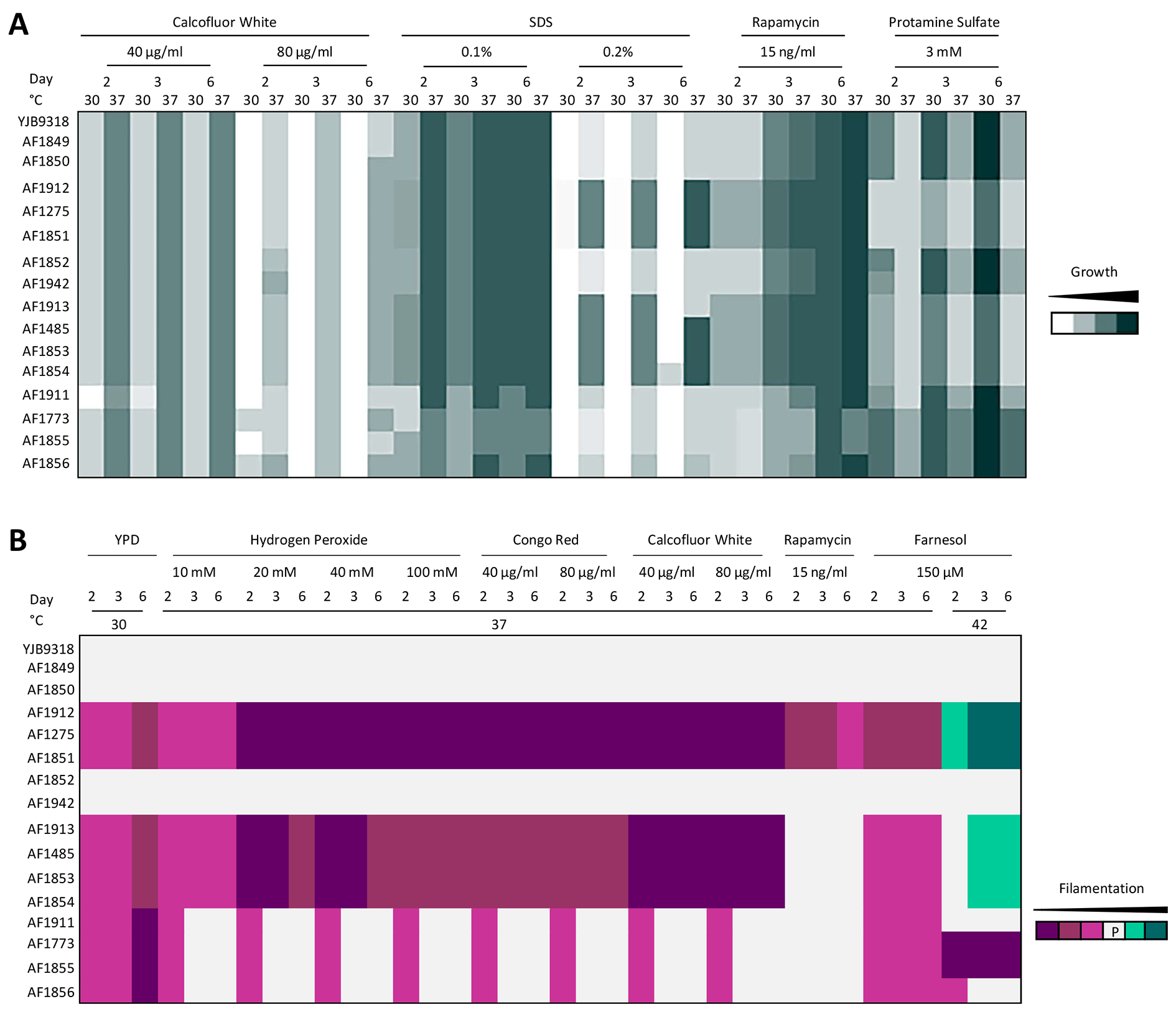

Supplement: S1 Fig — Shown are the conditions for which differences in growth (A) and spot morphology (B) were seen either between the progenitor and the trisomic strain(s) or between the different trisomic strains. Data is arranged by the day the plates were scored, the incubation temperature and growth medium. Strains and their derivatives are ordered as follows: progenitor, Chr6ABB, Chr6AAB, Chr5AAB. See S1 Table for strain information. (TIF) [file pgen.1008137.s001.tif]

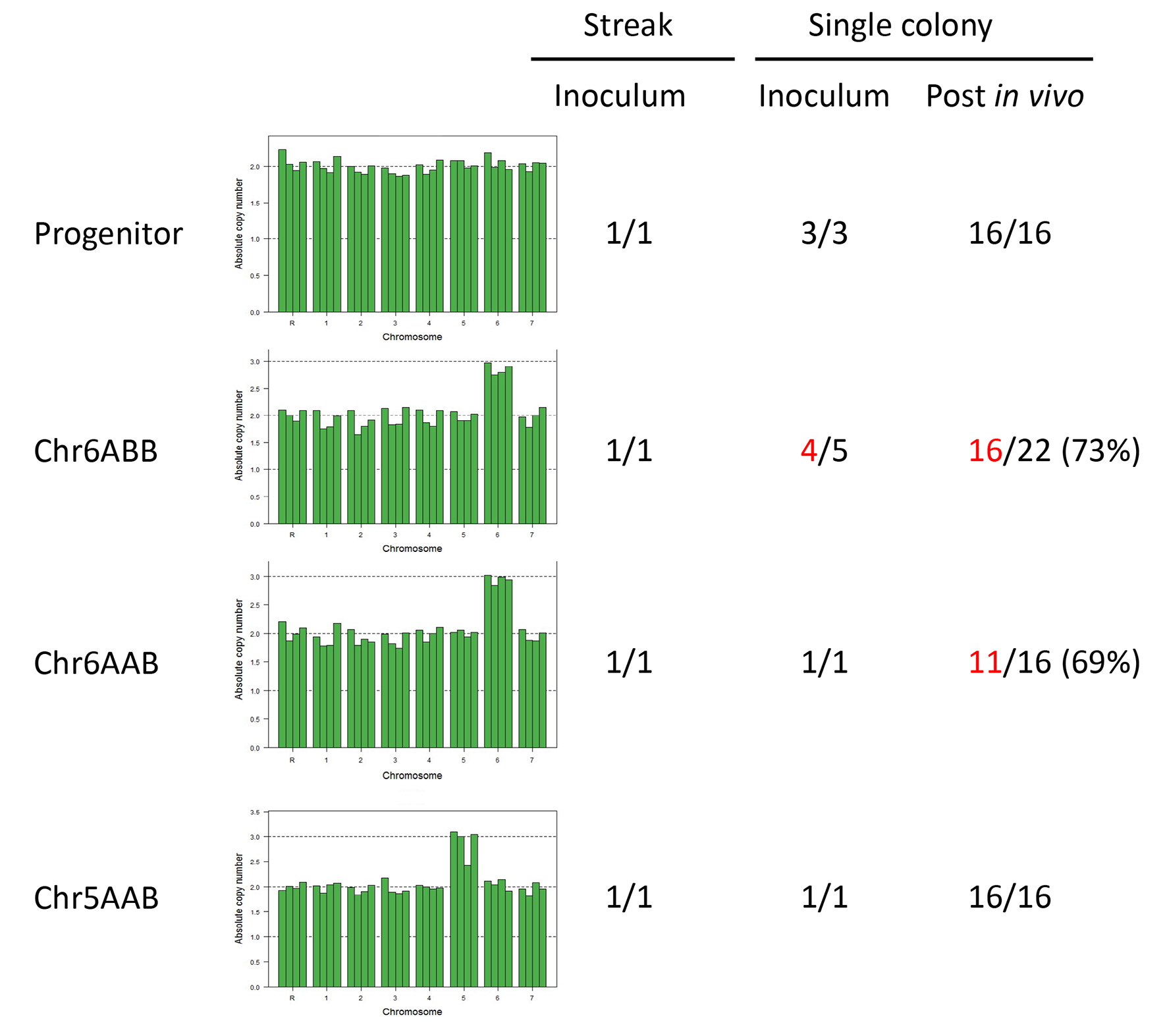

Supplement: S2 Fig — Ploidy genotypes were stably maintained for the parent and the Chr5AAB lineage but not for either Chr6 lineage. The ploidy genotype of the indicated strains was determined by qPCR for 4 markers along each of the 8 chromosomes. (TIF) [file pgen.1008137.s002.tif]

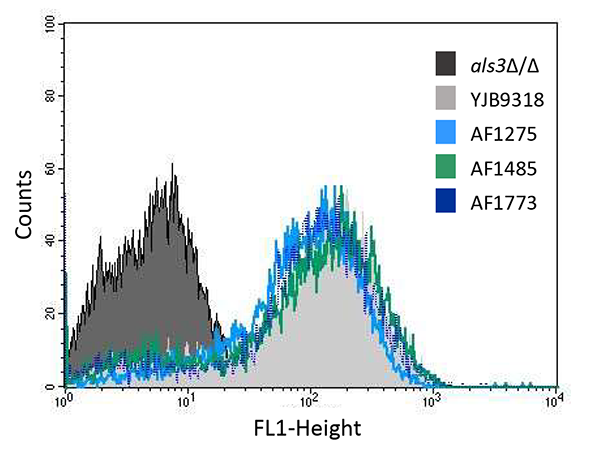

Supplement: S3 Fig — Flow cytometric analysis of progenitor (YJB9318), AF1275 (Chr6ABB), AF1485 (Chr6AAB), AF1773 (Ch5AAB), and an als3Δ/Δ delete strain as negative control. The histogram shows the results of analysis of 10,000 cells per strain. (TIF) [file pgen.1008137.s003.tif]
